# A Novel Synchronous MAC Protocol for Wireless Sensor Networks with Performance Analysis

**DOI:** 10.3390/s19245394

**Published:** 2019-12-06

**Authors:** Prasan Kumar Sahoo, Sudhir Ranjan Pattanaik, Shih-Lin Wu

**Affiliations:** 1 Department of Computer Science and Information Engineering, Chang Gung University, Taoyuan 33302, Taiwan; pksahoo@mail.cgu.edu.tw (P.K.S.); sudhirpattanaik.oca@gmail.com (S.R.P.); 2 Division of Colon and Rectal Surgery, Chang Gung Memorial Hospital, Linkou 33305, Taiwan; 3 Department of Computer Science and Engineering, National Institute of Science and Technology, Berhampur 761008, Odisha, India; 4 Department of AI Innovation Research Center, Chang Gung University, Taoyuan 33302, Taiwan; 5 Department of Cardiology, Chang Gung Memorial Hospital, Linkou 33305, Taiwan; 6 Department of Electrical Engineering, Ming Chi University of Technology, New Taipei City 24301, Taiwan

**Keywords:** wireless sensor networks, IEEE 802.15.4e, performance analysis, DSME, mesh topology

## Abstract

Synchronous medium access control (MAC) protocols are highly essential for wireless sensor networks (WSN) to support transmissions with energy saving, quality services, and throughput in industrial, commercial and healthcare applications. In this paper, a synchronous channel access mechanism is designed, where sensors can reserve the contention free data transmission slots in different available channels. To reduce the delay of data transmission among the nodes in the mesh topology, a linear programming problem (LPP) model is designed to select suitable relay nodes. Moreover, the performance of the proposed MAC is analyzed and our models are validated with simulation and analytical results. The results show that our proposed MAC protocol outperforms the IEEE 802.15.4e MAC mechanism in terms of throughput, reliability, delay, energy, packet drop rate and transmission success rate.

## 1. Introduction

A wireless sensor network (WSN) consists of a large number of autonomous sensor nodes, which are mostly used for environmental monitoring, military surveillance, smart buildings, health care and industrial applications. Deployment of WSNs is suitable for harsh environments, where human interventions are limited. WSNs are constructed through the interconnection of wireless sensors, where each node not only sends its own data but also relays data from other neighboring nodes. The main advantages of WSNs are that data can be transmitted from different sensor nodes simultaneously by spatial reuse of a channel and can always have alternative options to send the data, even if some sensor nodes have failed. Thus, the network spectrum can be reused with high reliability. In many applications of wireless sensor networks, WSNs are considered as an emerging technology.

In WSNs, sensor nodes communicate with other sensors through a wireless medium, where channel conditions oscillate between good and poor. However, when heterogeneous nodes in WSNs share the same ISM band, the network performance is degraded in single channel operations due to interference. Besides, hidden and exposed terminal problems [1] are most important issues in single channel communication environments. Hence, single channel communication cannot provide reliable transmissions. Multichannel communications can enhance reliable transmissions, alleviate hidden/exposed terminal problems, minimize the network interference and support parallel data communication, as a result of which network throughput can be increased substantially. In order to access a channel in a WSN, synchronous and asynchronous medium access control (MAC) protocols are used. In the synchronous protocol, all nodes wake up at the same time, while in the asynchronous protocol nodes have different wake up times. Thus, the synchronous protocol is most suitable for real time data transmission as it does not require any extra negotiation for data exchange.

The important parts of communication by a node in WSNs are network discovery, association and successful transmission of requests. Before any communication in the network, a node performs a random backoff and clear channel assessment (CCA). During the backoff process, nodes deployed over the network cannot identify whether the channel is currently idle or not. The tagged node found the channel busy during its CCA when the remaining time of an ongoing transmission is greater than the chosen random backoff. Hence, the tagged node goes for the next CCA after additional random backoff. Based on the existing MAC mechanism of IEEE 802.15.4 [2], two times consecutive CCA operation is required before any data transmission. However, performing CCAs twice is inefficient as it requires more delay and energy consumption than performing it once. Energy is the main constraint for the battery powered sensor devices and therefore a suitable channel access mechanism should be designed to optimize the energy usage in WSNs.

In WSNs, discovery of neighboring nodes is a fundamental task, where a new node initiates the channel scan over a given list of channels before joining the network. A coordinator in a network initiates and manages the whole network. Nodes in beacon-enabled networks rely on passive scans in different channels to find a coordinator for association. In passive scans, a to-be-associated node only listens to the medium for beacons and the latency of discovery depends on the coordinator’s beacon interval (BI) and the available channels of the WSN. The node is not aware about the particular channel number as well as the beacon broadcast interval used by the coordinator. Hence, the node has to scan all the channels with all possible beacon orders, which is a time consuming process. An active scan, as suggested in IEEE 802.15.4e standard [3], uses the beacon request command to extract the beacon from a coordinator. The to-be-associated node transmits the beacon request command in each channel one by one and waits on that channel for the most certain threshold time to receive the information.

In WSNs, data transmissions in the network are mainly sink oriented. When a node fails due to lack of energy, a new link is established for the data transmissions. A node that wants to send data to the sink simply sends to its one hop neighbor close to the sink. The coordinator/neighbor, upon receiving data from its children, sends to the next hop along with its own data. Hence, selection of the relay node is a crucial factor for the data routing to the sink from a node in the WSN, which can improve the throughput. In order to mitigate the problems related to the latency, reliability, throughput and energy consumption in a synchronous WSN, novel channel access mechanisms and performance analysis models are proposed. Accordingly, the main goals of our work can be summarized as follows.

- The limitations of the channel access mechanism of IEEE 802.15.4e deterministic and synchronous multichannel extension (DSME) MAC used for the WSN in multi-channel synchronous environments are analyzed.
- A new channel access mechanism is designed to reduce the energy consumption during communications in any control channel.
- A linear programming (LP) model is designed to select the suitable relay nodes efficiently.
- Analytical models are developed to evaluate the delay, throughput, reliability and packet drop rate of the proposed MAC as compared to the DSME MAC of IEEE 802.15.4e.

The rest of the paper is organized as follows. Related works are given in Section 2 and our proposed network model and methods are elaborated on in Section 3. Performance analyses of various network parameters are given in Section 4. Model validation and performance evaluation of our proposed protocols are presented in Section 5. The overall concluding remarks are made in Section 6.

## 2. Related Work

Low latency, data reliability and drop rate are various critical requirements for industrial and commercial applications. WSNs are the most used network in different applications such as industrial automation and smart building. However, the performance degradation of WSNs is a common challenge due to the erroneous nature of the wireless medium. To improve the performance of WSNs, authors in [4] designed a time division multiple access (TDMA) based reliable multicast transmission protocol, which can improve the reliability, and one modified channel access mechanism is proposed in [5] to enhance the throughput of the network. Authors in [6] propose a guaranteed time slot (GTS) size adaptation algorithm to use the guaranteed time slots optimally. Authors in [7,8] have studied the performance of WirelessHART, which is a centralized network management architecture for the data communication to optimize the delay, reliability and real-time performance. However, they have studied only the periodic data flows and have considered the TDMA based approach, where each slot duration allows exactly one transmission. Authors in [9] introduce a new cooperative MAC protocol that reduces the energy consumption based on the residual energy of relay node. Authors in [10] have proposed a relay selection technique based on the neighbors, remaining battery power, quality of the link and the success rate. However, the selection of the relay node does not consider the arrival or service rate and buffer capacity, which are important factors for the low latency data transmission. For deterministic performances in different applications, slotted MAC is used for communication. Hence, authors in [11] have investigated the superframe structure of the slotted MAC through mathematical models to minimize the latency based on the packet generating rates.

Randomness can lead to a considerable probability of collision. Hence, authors in [12] propose a scheduling method that eliminates the collisions of the beacons. A robust channel allocation mechanism is proposed in [13] by exploiting the partially overlapped channels for the directional multi-channel wireless mesh networks. An interference problem arises when multiple wireless nodes work in the same industrial, scientific and medical (ISM) band, due to which a significant amount of packet loss occurs that degrades the expected performance. Moreover, a node goes for a random backoff and CCA for every collision/transmission failure. However, if a node can estimate the ongoing transmission duration during the CCA, it can take the appropriate backoff duration to reduce the energy consumptions. Authors in [14] propose an extended shortcut tree routing protocol to improve the network lifetime. However, they have not considered the interference issues among the nodes. Authors in [15] have designed a priority-aware multi-channel adaptive framework to increase the reliability of the network, which is suitable for the tree topology only. Multichannel MAC supports the parallel transmission and reduces the interference. In order to reduce the interference problems, authors in [16] introduce a dynamic MAC protocol to find the best communication channel to enable the coexistence between wireless nodes using the same ISM spectrum. In order to overcome the interference between WSN and WLAN, authors in [17] design a lightweight cognitive MAC (COG-MAC) for WSN under the severe interference of WLAN. However, a substantial amount of interference can be mitigated through multichannel communication. Hence, authors in [18] propose a distributed link-based MinMax channel allocation protocol, the objective of which is to minimize the maximum interference experienced by any transmission link in a WSN. Clock synchronization between the transmission nodes plays a crucial role in reducing energy consumption in a network. Therefore, authors in [19] designed a synchronization scheme based on a dynamic superframe for an underground mining network. A contention-free safety message broadcasting and data transmission protocol is designed in [20] for vehicular ad hoc networks (VANETs). However, the proposed protocol is not suitable for the energy constraint wireless sensors. In order to save the power, authors in [21] designed a novel energy efficient adaptive power control protocol to adjust the transmission power level based on the feedback information from the base station. However, there is higher packet loss ratio in the network. Failure of some nodes in WSN can partition the network into different segments and network connectivity is lost. Authors in [22] propose a distributed energy efficient solution for multi-channel allocation based on the routing that reconnects the network after a node fails. A low duty cycle reduces energy consumption in WSNs. However, synchronous duty cycled MAC protocol achieves better energy efficiency than the asynchronous one. Hence, authors in [23] propose an analytical model to evaluate the network performance metrics for Sensor Medium Access Control (SMAC). The impact of inserting a MAC-level finite buffer on the performance of WSNs has been studied in [24]. However, these works mainly focus on star topology.

Performance evaluation of cross-layer IEEE 802.15.4/4e/4g LR-WPAN is studied in [25]. Authors in [26] design a cross-layer model of the MAC and physical layers for unslotted IEEE 802.15.4 networks. A comparative study between IEEE 802.15.4e LLDN and IEEE 802.15.4 slotted carrier sense multiple access with collision avoidance have been analyzed in [27] under ideal channel conditions, which are not realistic. The performance of single and multi-hop networks under unslotted IEEE 802.15.4 standard MAC protocol is analyzed in [28]. However, the authors do not talk about how to choose the next hop node for data transmission in order to increase the network lifetime. A carrier sense multiple access with collision notification (CSMA/CN) protocol is proposed in [29] to approximate the carrier sense multiple access with collision detection (CSMA/CD) for the wireless network, which increases reliability though the energy consumption is high. To handle bursty data with high reliability, authors in [30] propose a receiver-centric MAC protocol to reduce collisions and to achieve high throughput.

IEEE 802.15.4e standard introduces the deterministic and synchronous multichannel extension (DSME) MAC to support nodes for low data rate, low power and short-range communication, which is capable of addressing the emerging needs of different applications. The DSME MAC mode extends the single channel operation of IEEE 802.15.4 [2] to multichannel operation for data transmissions to reduce the effects of mutual interferences due to heterogeneous nodes. A new DSME MAC protocol is designed in [31] to avoid the channel being busy due to acknowledgment packet transmission without any additional CCA. Analytic approach is designed in [32] to investigate the queue-size transient distribution. Performance evaluation of DSME-enabled IEEE 802.15.4e standard WSNs in terms of frame error rate and aggregate throughput have been studied in [33] for star topology, but not for mesh topology. The authors of [34] analyze the throughput and energy consumption of the DSME MAC protocol of the IEEE 802.15.4e standard. Though DSME MAC supports synchronous multichannel communication that enhances the network throughput and reduces latency, it cannot avoid beacon collision in dense networks. The authors in [35] propose an energy efficient enhanced multichannel MAC protocol to reduce latency. The authors in [36] proposed one routing protocol of the wireless multi-hop mesh networks considering multiple paths and transmission channels at the same time, to achieve the quality of service. However they did focus on the selection of the intermediate relay node to enhance the network lifetime. To handle variable traffic pattern, the authors in [37] propose a hybrid MAC, which operates in CSMA mode at low traffic TDMA mode. Considering the prioritization during channel access, the authors in [38] propose a mechanism where, during contention, the backoff period ranges for each traffic class for IEEE 802.15.4.

Comparisons of earlier works are shown in Table 1. However, from the survey of the related literature, it is observed that most of the works on performance analysis are based on the IEEE 802.15.4 MAC with star topology or single channel. To the best of our knowledge, though few works analyze the performance of the wireless mesh sensor network, none of the work evaluates the reliability and latency of the deterministic and synchronous multichannel extension (DSME) MAC of IEEE 802.15.4e used for the WSN in a multi-channel synchronous environment. We design here the performance analysis models as follows.

## 3. Proposed Synchronous MAC Protocol

In this section, a synchronous MAC protocol is designed for the WSNs. To reduce the power consumption for communications in the control channel, a new MAC protocol is designed and the performance analysis of the proposed MAC is developed in the next section. The system model of our proposed protocol can be designed as follows.

### 3.1. Network Model

**Figure 1:**
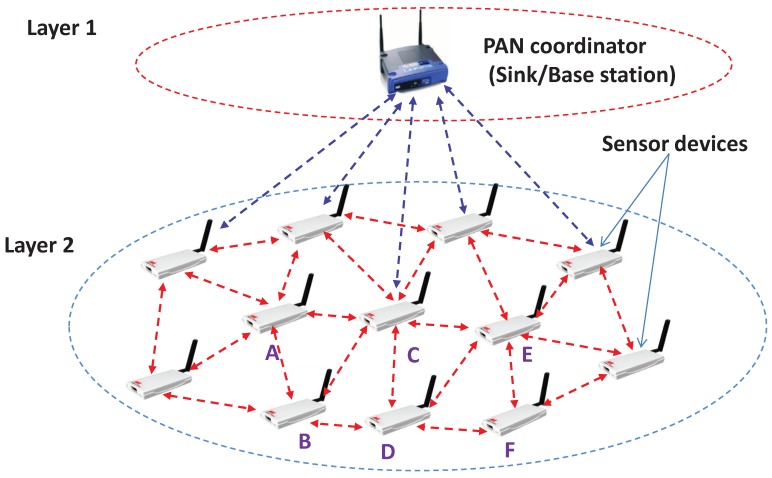
The two layered wireless sensor network.

Consider a wireless sensor network deployed over a certain region, which forms a two tier mesh topology as shown in Figure 1. The wireless sensor nodes in the second layer form a mesh topology to collect the data and to forward them to the next higher layer, i.e., to the network coordinator or the sink through two hops. It is to be noted that all nodes/coordinators in the network are refereed to as nodes throughout the paper and they use the carrier sense multiple access with collision avoidance (CSMA/CA) channel access mechanism to send or receive data. Let, *N* be the number of nodes present within the carrier sensing range of one node in the WSN.

**Figure 2:**
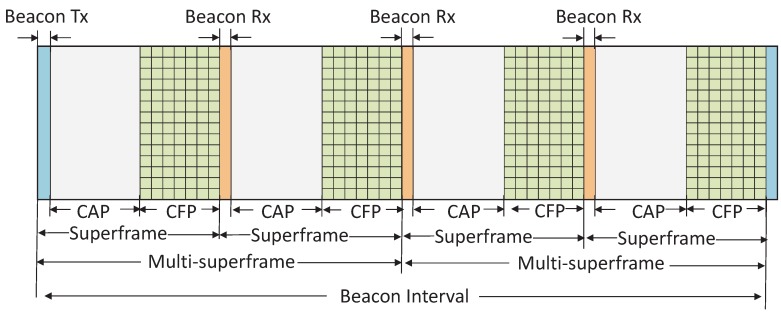
Deterministic and synchronous multichannel extension (DSME) superframe structure.

Let us assume that each node uses a fixed number of multiple channels and a multi-channel superframe (MCS) structure to compete with other nodes for accessing those channels to transmit the data. Out of the multiple channels, one channel is used as the control channel and the rest are used as the data channel. The control channel is used only for beaconing by the devices in the network. For simplicity, the superframe structure can be considered as the DSME mode of the IEEE 802.15.4e standard, as shown in Figure 2. The BI comprises several multi-superframes without inactive parts. Each multi-superframe has a beacon slot at its beginning and consists of several superframes with contention access periods (CAPs) and contention free periods (CFPs). These CAPs and CFPs are used for the control and data packet transmission, respectively. It is to be noted that each superframe consists of 16 time slots of equal duration and the first slot of the first superframe present in the MCS is reserved for the PAN coordinator to broadcast the beacon. The number of beacon slots is equal to the number of superframes present in an MCS. For example, as shown in Figure 2, there are four beacon slots as the MCS consists of four superframes. Only one beacon slot is used by one node to broadcast the beacon and the rest of the beacon slots are used to receive the beacon frames from other nodes.

It is to be noted that the beacon interval comprises several multi-superframes without inactive parts and each multi-superframe has a beacon slot at its beginning. One beacon slot is used by the PAN coordinator to broadcast the beacon and the rest of the beacon slots are used to receive the beacon frames from other nodes. The beacon broadcast by the PAN coordinator is used for the purpose of synchronization with the superframe structure. It is also used for the re-synchronization of the nodes which are in the power saving or sleep mode. It is to be noted that a node is usually equipped with a half-duplex transceiver, i.e., it cannot listen when it transmits. Hence, the channel coordination is a crucial issue for solving the deafness problem. To handle these problems, the nodes use the CAP period to be associated with the other nodes or to send the aperiodic data or reserve the contention free time slots using the slotted CSMA/CA. A node during CAP defers transmission if the remaining slot time is not big enough to transmit the request. The CFP period supports the multichannel communication and is used for the periodic data transmission by the allotted nodes without contention. Besides, a group acknowledgement (GACK) option is considered to provide a retransmission opportunity of a data frame within the same superframe if it failed in its GTS transmission. The group acknowledgement also improves the efficiency of the communication as the acknowledgement for multiple data frames is aggregated in a single ACK frame.

### 3.2. New Channel Access Mechanism

It is to be noted that the CSMA/CA mechanism in WSN normally uses the MAC mechanism of IEEE 802.15.4, where one backoff unit/slot in the IEEE 802.15.4 standard is of 20 symbols. CCA requires eight symbols time to know the status of the channel, and the turnaround time (Tx-Rx or Rx-Tx) is 12 symbols. If the remaining symbol of the data frame at the end of backoff slot is less than eight, the receiver transmits the acknowledgement in the very next backoff slot. Otherwise, it has to transmit into the next two backoff slots. Our intention is to avoid this situation by sending a known signal to make the data channel busy so that other nodes can know about it and go for the appropriate sleep period based on its average packet duration time to avoid the energy consumption due to unnecessary CCA. Accordingly, we propose to insert three known signal sequences at the start of the data, end of the data and start of the acknowledgement frames if the remaining symbol of the data frame at the end of the backoff slot is greater than eight. Otherwise, insert only two known signal sequences at the start of the data and acknowledgement frames. While accessing the data channel, the nodes that receive these known signals during the CCA calculate the appropriate sleep duration and random backoff time to avoid further unnecessary CCAs based on the correlation value of the known signal and received signal.

**Figure 3:**
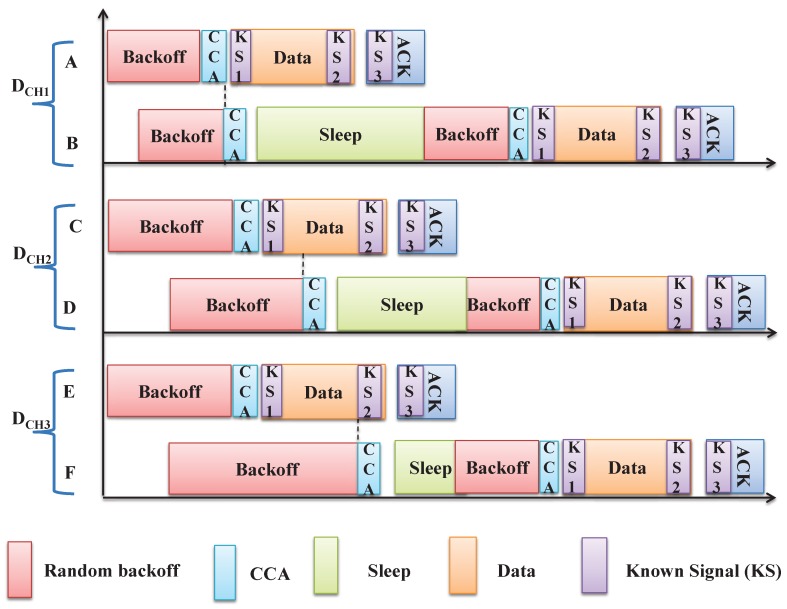
Proposed multiple data channel access mechanism.

For example, as shown in Figure 3, let us consider three data channels to be accessed by the nodes A,B,C,D,E and *F*. The channel access in the data channel $D_{CH1}$ is successful for the device *A* and it starts transmitting the packet and waits for the acknowledgement. The reception of the corresponding ACK is interpreted as the successful packet transmission. As shown in Figure 3, the device *B* finds the channel busy during the CCA in the $D_{CH1}$, just as device *A* starts the data transmission. Device *B* receives one signal during its CCA and knows if the cause of the channel is busy during the CCA. Hence, it consequently adopts the sleep state for the average duration of the data and acknowledgement period. Similarly, the device *D* finds the channel busy due to the transmission of data by the device *C* in the data channel $D_{CH2}$. Here, device *D* does not know the exact average remaining time for the ongoing communication and therefore goes to the sleep state for a random duration of the average packet communication period. As shown in Figure 3, the device *F* performs its CCA at the last backoff slot of the data packet duration of the device *E* in the data channel $D_{CH3}$ and finds the channel busy. Hence, it switches to the sleep state for the duration of the acknowledgement period to save energy. Similarly, a device may find the data channel is busy due to the acknowledgement and switches to the sleep state for the remaining duration of the acknowledgement.

It is to be noted that the nodes can easily detect a signal by comparing the amplitude of a correlation value against the present threshold without demodulating an exact symbol in random noise. Let X[n] be the complex number representing the *n*th transmitted symbol and Y[n] be the complex number representing the *n*th received symbol. Then

(1) $$\begin{array}{cc} \;\; & Y[n]=H*X[n]+\omega [n] \end{array}$$

where, *H* is a complex number representing the channel coefficient between the transmitter and the receiver, and ω[n] is the random noise. The cross correlation for the known symbol pattern of length *L* in the received signal *Y* at a shifted position *p* is given by the following equation, as in [29],

(2) $$\begin{array}{cc} \;\; & C\left(KS,Y,p\right)=\sum_{i=0}^{L}\bar{KS[i]}+Y\left[i+p\right], \end{array}$$

where $\bar{KS[i]}$ is the complex conjugate of KS[i]. The correlation coefficient C(KS,Y,p) is low when the known signal KS is not present in *Y*. Taking a signal threshold $\left(S_{th}\right)$ for the correlation coefficient, we can detect the presence of the known signal. The value of the cross-correlation is above the threshold when the known signal is aligned to the same features in the received signal.

As shown in Algorithm 1, we consider a node that tries to transmit a request during the CAP period of the DSME superframe following the CSMA/CA procedure. Accordingly, we initialize the variables NB, CW, BE and RT. NB is the number of backoffs while attempting the current transmission. CW is the number of backoff periods to be cleared before transmission. BE, σ and RT are the backoff exponent, unit backoff duration and maximum number of retransmission attempts, respectively. Then, the MAC layer delays for a random backoff period $\left(R_{b}\right)$ in the range of $\left[0;2^{BE}-1\right]$ units. When the backoff period counts down to zero, a node performs a CCA if the remaining time $\left(R_{CAP}\right)$ in the CAP period is enough to transmit the requested packet after CCA. Let the duration required to perform the CCA and average data transmission be $T_{CCA}$ and $T_{D}$, respectively. It is proposed that a node can transmit the request if the channel is idle during its CCA. For example, as shown in Figure 3, the channel access is successful for the node *A* for which it starts transmitting the packet and waits for the acknowledgement.

The reception of the corresponding ACK is interpreted as the successful packet transmission. The node finds the channel busy during the CCA if any other node is transmitting during the tagged node’s CCA. As shown in Figure 3, there are four cases where a node can find the channel busy. Upon knowing the cause of the channel being busy during the CCA, the tagged node goes for an appropriate sleep period based on its average packet duration time and then performs the random backoff before trying for the next CCA in that data channel. The maximum value of NB is maxMacBackoff $\left(MAX_{NB}\right)$ and the value of NB is incremented for each channel access failure. If the value of NB exceeds the value of $MAX_{NB}$, it is treated as a channel access failure and the packet is discarded. The maximum number of retransmissions due to collision or channel error is limited to a value a MaxFrameRetries. Each transmission failure increases the value of retransmission (RT) by one. If the value of RT is more than a MaxFrameRetries $\left(MAX_{RT}\right)$, the packet is also discarded. The procedure continues until the tagged node attains the maximum number of channel access failures.

**Algorithm 1** Proposed new channel access mechanism. **Require:**
$NB=0,CW=CW_{0},BE=BE_{min},RT=0,\sigma =20\;symbol,T_{CCA}=8\;symbol$, average packet duration time $T_{D}$. **Ensure:** Transmission success/failure.
 1:
**if Beacon is not received in the control channel then**
 2:    **Wait for the beacon in the control channel**; 3:
**else**
 4:    **Switch to data channel and generate a random backoff $R_{b}$ = random(0, CW)×σ duration**; 5:    **if** $\left(R_{CAP}\right)\leq \left(R_{b}+T_{CCA}+T_{D}\right)$ **then** 6:        Wait for next superframe and go to step 1; 7:    **else** 8:        Performs CCA after $R_{b}$ duration and receives signal *Y* during this CCA; 9:        **if** Channel found busy **then**10:           **if** $C\left(KS_{1},Y,p\right)\geq S_{th}$ **then**11:               Switch to sleep state for $T_{D}+T_{A}$ duration;12:           **else if** $C\left(KS_{2},Y,p\right)\geq S_{th}$ **then**13:               Switch to sleep state for $T_{A}$ duration;14:           **else if** $C\left(KS_{3},Y,p\right)\geq S_{th}$ **then**15:               Switch to sleep state for $R_{A}$ duration;16:           **else**17:               Switch to sleep state for $Random(0,T_{D})$;18:           **end if**19:           NB=NB+1, $BE=min(BE+1,BE_{min})$;20:           **if** $NB>MAX_{NB})$ **then**21:               Channel access failure;22:           **else**23:               Go to step 4;24:           **end if**25:        **else**26:           **Start transmission in the data channel**;27:           **if** ACK is not received **then**28:               RT=RT+1;29:               **if** $RT\leq MAX_{RT}$ **then**30:                   Go to step 4;31:               **else**32:                   Transmission failure;33:               **end if**34:           **end if**35:        **end if**36:    **end if**37:
**end if**



### 3.3. Selection of Relay Node

The nodes of a WSN are normally powered by batteries that cannot be replaced and replenished. In the case of power failure, data collected and routed through those nodes are lost. It is desired that the nodes should optimize the power consumption to extend the network lifetime. Moreover, selection of relay nodes is another important factor that needs to be considered. In this section, we discuss how to choose the next hop relay nodes to extend the network lifetime. Let $S_{1},S_{2},\ldots ,S_{N}$ be the *N* number of nodes present within the carrier sensing range of a node $S_{0}$. Hence, the node $S_{0}$ transmits the data to the sink through these nodes. By choosing the suitable nodes with a good signal to noise ratio (SNR), we can reduce the number of retransmissions, which will ultimately reduce the energy consumption and increase the network lifetime. Hence, it is proposed to choose those neighbor nodes as the relay nodes, which are one hop away from the sink/base station with higher remaining energy and value of the SNR greater than a certain threshold.

Assume that there are *N* number of such nodes present within the carrier sense range of one node. Let $\lambda_{i}$ and $\mu_{i}$ be the total data arrival rate (packets/second for both sensed and received) and successful data transmission rate (1/T) by the node $S_{i}$, respectively, while *T* is the time period from the packet arrival time to the time the packet is successfully transmitted. Assume that each node first buffers the received packets and then transmits them to the sink. Each node may have buffer constraints. Let, $BF_{i}$, $RE_{i}$, $SNR_{i}$ be the buffer capacity, residual energy and SNR value of the *i*th node, respectively. Assume that, for each such node $S_{i}$, $\lambda_{i}$, $\mu_{i}$, $BF_{i}$, $RE_{i}$ and $SNR_{i}$ for i=1,2,…,N are known to the node $S_{0}$. For example, as shown in Figure 1, it is assumed that the node *D* with data arrival rate $\lambda_{D}$ has the information about the prior data arrival rate, remaining energy, data transmission rate, SNR value and buffer capacity of nodes *A* and *B*. Hence, node $S_{0}$ can calculate the utilization rate of these *N* intermediate nodes, which is $\rho_{i}=\frac{\lambda_{i}}{\mu_{i}}$ for i=1,2,…,N. Let us assume that a node can buffer the maximum *l* number of packets. Hence, it will just drop the remaining packets if more than *l* number of packets arrive at a node. Due to such restrictions, the sensor node $S_{0}$ should carefully choose the number of packets to be transmitted to these *M* nodes. Let $P_{k,i}$ be the probability that there are *k* number of packets in the buffer of the $i^{th}$ node, where k=0,1,2,…,l and i=1,2,3...N. Then

(3) $$\begin{array}{cc} P_{k,i} & =\frac{\rho_{i}^{k}}{\sum_{l=0}^{k}\rho_{i}^{l}}. \end{array}$$

Therefore, the effective arrival rate of these *N* nodes should be selected in such a way that it should not exceed the buffer size. Hence, the effective arrival rate of these nodes should be modeled as follows so that all data packets can be transmitted.

(4) $$\begin{array}{cc} \lambda_{e,i} & =\lambda_{i}\times \left(1-P_{l,i}\right)\;\;for\;1\;\leq \;i\;\leq \;N \end{array}$$

The problem is now what fraction $X_{i}$ of the data arrival rate $\lambda_{0}$ of the node $S_{0}$ should be allocated to the *i*th node, i=1,2,…,N, so that the longer lifetime of the network with low latency can be achieved. For this, a weight value for each node is introduced considering the remaining energy, data transmission rate and current buffer status. The weight of the *i*th node is calculated as

(5) $$\begin{array}{cc} \omega_{i} & =a_{0}\mu_{i}+a_{1}RE_{i}+a_{2}BF_{i} \end{array}$$

where $\sum_{j=0}^{2}a_{j}=1$ and $a_{j}\geq 0$. Under these assumptions, the optimized allocation, *Z*, to a node can be expressed as follows.

(6) $$\begin{array}{c} \;Max\;Z\;=\sum_{i=1}^{N}\;\lambda_{i}+\lambda_{0}X_{i}\\ \mathrm{subject}\;\mathrm{to}\\ \lambda_{i}+\lambda_{0}X_{i}\leq \mu_{i}\\ X_{i}\;\leq \;\frac{\omega_{i}}{\sum_{i=1}^{N}\omega_{i}}\\ \sum_{i=1}^{N}X_{i}=1\\ X_{i}\geq 0,\;1\;\leq \;i\;\leq \;N \end{array}$$

This proposed LPP model evaluates the optimal allocation for each node so that the energy consumption can be balanced with a lower packet drop rate.

### 3.4. Analysis of Proposed MAC

Let us consider a WSN in which *N* number of nodes are associated with a coordinator. The CSMA/CA mechanism reduces collisions by using binary exponential backoff. Let

$$b_{i,j}=\lim_{t\to \infty }P\left\{s\left(t\right)=i,c\left(t\right)=j\right\},$$

where s(t) and c(t) represent stochastic processes for the backoff stage *NB* and backoff counter *CW*, respectively, and $j\in \{-1,\ldots ,W_{i}-1\}$, $W_{i}=2^{min(i+macMINBE,macMAXBE)}$ for i∈{0,…,m}. BE is the backoff exponent which determines the number of backoff periods and the time *t* corresponds to the system time. $b_{i,-1}$ represent the states corresponding to the channel access for i∈{0,…,m} after the backoff counter reaches zero with sufficient time remaining in the CAP period for transmission. $b_{0,-2}$ and $b_{-2,0}$ represent the transmission and idle states, respectively. The node will be in the transmission state if the channel is found idle during its CCA. However, it remains in the idle state if it has no request packet to transmit. Descriptions of different symbols and notations are summarized in Table 2.

**Figure 4:**
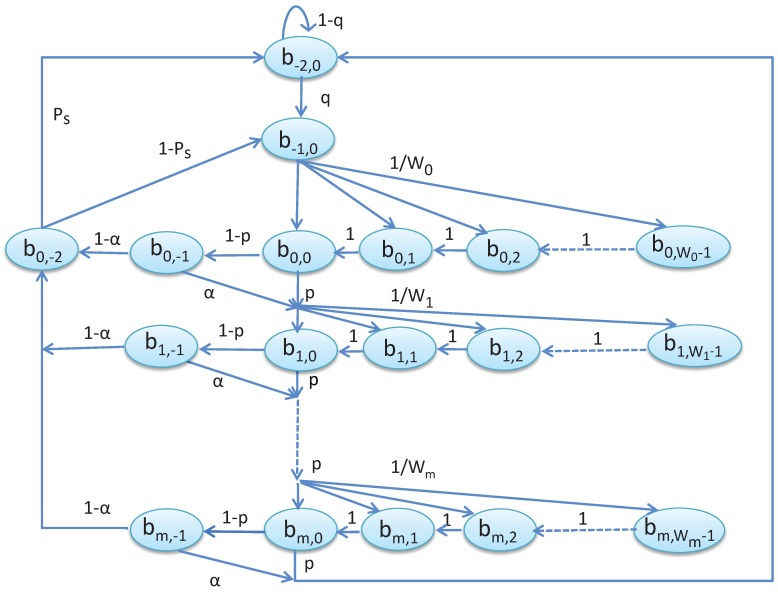
Markov chain model for the proposed carrier sense multiple access with collision avoidance (CSMA/CA) mechanism.

Let, *q* be the probability that the node has a request packet. The node moves to the active state $b_{-1,0}$ when it has a request packet and performs the random backoff before it proceeds to the state $b_{0,k}$, where $k\in \{0,1,2,\ldots ,W_{0}-1\}$. The state $b_{i,j}$ represents the backoff states at different backoff stages, where i∈{0,…,m} and $j\in \{-1,\ldots ,W_{i}-1\}$. Let us assume the probability that a channel is busy during the CCA is α and that insufficient data transmission time after the random backoff in the remaining CAP is *p*. A node moves to the transmission state if the channel is found to be idle during CCA and starts to attempt to transmit requests. Let transmission successful probability be $P_{S}$. Based on the proposed Markov chain model as given in Figure 4, we get the followings.

The transition probabilities used to derive the steady state probabilities are given as follows.

(7) $$\begin{array}{cc} P\{b_{i,k}|b_{i,k+1}\} & =1\;\;for\;0\leq i\leq m\;and\;0\leq k\leq W_{i}-1. \end{array}$$

(8) $$\begin{array}{ll} P\{b_{i,k}|b_{i-1,0}\} & =\frac{p+(1-p)\alpha }{W_{i}}\\ & \;for\;0\leq i\leq m\;and\;0\leq k\leq W_{i}-1. \end{array}$$

(9) $$\begin{array}{cc} P\{b_{-2,0}|b_{m,0}\} & =\left(p+\left(1-p\right)\alpha \right)+\left(1-p\right)\left(1-\alpha \right)P_{S}. \end{array}$$

Equation (7) derives that the nodes decrement the backoff counter with probability 1. Equation (8) deduces the channel busy probability during CCA and selects the next backoff state. Equation (9) derives the probability of going back to the idle state. Based on these transition probabilities the steady state probabilities can be derived as follows. The steady state probability of entering into the next backoff state by a node is

(10) $$\begin{array}{ll} b_{i,k} & =\frac{W_{i}-k}{W_{i}}\left(\left(\left(1-p\right)\alpha +p\right)b_{i-1,0}\right);\\ & \;\;\;for\;1\leq i\leq m\;and\;0\leq k\leq W_{i}-1. \end{array}$$

A tagged node starts transmission when the channel is found to be idle during CCA. It enters into the initial backoff state if the transmission is successful and has a new data packet to send or transmission failure occurs without exceeding the retransmission limits. Hence, the steady state probability can be derived as

(11) $$\begin{array}{c} \begin{array}{cc} b_{0,k} & =\frac{W_{0}-k}{W_{0}}(\left(1-p\right)\left(1-\alpha \right)\left(\left(1-P_{S}\right)+qP_{S}\right)\sum_{i=0}^{m}b_{i,0}\\ \; & +\left(\left(1-p\right)q\alpha +pq\right)b_{m,0}). \end{array} \end{array}$$

A node starts channel assessment if the remaining CAP duration after a random backoff period is enough for data transmission. Hence the corresponding steady state probability can be derived as follows.

(12) $$\begin{array}{cc} b_{i,-1} & =\left(1-p\right)b_{i,0}. \end{array}$$

The node starts transmitting data after accessing the channel successfully in the *i*th backoff state. Hence, the corresponding steady state probability can be deduced as

(13) $$\begin{array}{cc} b_{0,-2} & =\left(1-\alpha \right)\sum_{i=0}^{m}b_{i,-1}. \end{array}$$

A node enters the idle state after successfully transmitting the data or it has no packet to transmit. Hence, the corresponding steady state probability is

(14) $$\begin{array}{cc} b_{-2,0} & =\frac{1}{q}\left(P_{S}b_{0,-2}+\left(\left(1-p\right)\alpha +p\right)b_{m,0}\right). \end{array}$$

A node enters into the active state when it has to transmit the unsuccessfully transmitted packet or a new packet. Hence, the corresponding steady state probability can be derived as

(15) $$\begin{array}{cc} b_{-1,0} & =\left(1-P_{S}\right)b_{0,-2}+qb_{-2,0}. \end{array}$$

We can get the following equation based on the total probability rule.

(16) $$\begin{array}{cc} \sum_{i=0}^{m}\sum_{j=0}^{W_{i}-1}b_{i,j}+\sum_{i=1}^{m}b_{i,-1}+\sum_{k=1}^{2}b_{-k,0}+b_{0,-2} & =1. \end{array}$$

We can get all states’ probabilities by solving these equations. Then the probability ϕ that a node attempts carrier sensing for the first time can be calculated as follows.

(17) $$\begin{array}{c} \phi =\sum_{i=0}^{m}b_{i,0}\left(1-p\right). \end{array}$$

## 4. Performance Analysis

The probability of the bit error rate $P_{b}\left(\zeta \right)$ for the transceiver of IEEE 802.15.4 radios is calculated [2] as given in the following equation, where ζ is the signal-to-interference-plus-noise ratio (SINR).

(18) Pb(ζ)=815116∑k=216(−1)k16ke20ζ(1k−1)

The different packet lengths are provided in Table 3. Then, the probability that these packets are received successfully can be given as follows.

(19) $$\begin{array}{c} P_{i}={\left(1-P_{b}\left(\zeta \right)\right)}^{L_{i}};\;\;1\leq i\leq 10. \end{array}$$

The different packet generation probabilities are given in Table 3; let α be the probability that the CCA is busy. It is to be noted that the CCA will be busy during the transmission of different packets as described in Table 3. Let $\alpha_{1}$ through $\alpha_{9}$ be the probability that the node is busy during the CCA due to the transmission of the corresponding packets, which can be generalized as follows.

(20) $$\begin{array}{c} \begin{array}{cc} \alpha_{i} & =\left\{\begin{array}{c} L_{i}q_{i}\left(1-{\left(1-\phi \right)}^{N-1}\right)\left(1-\alpha \right);\\ \;\;\;\;\;\;\;\;\;\;\;\;\;\;for\;\;1\leq i\leq 4\\ L_{i}q_{i-4}\left(1-{\left(1-\phi \right)}^{N-1}\right)\left(1-\alpha \right)P_{i-4};\\ \;\;\;\;\;\;\;\;\;for\;\;5\leq i\leq 8\\ L_{i}q_{i-7}\left(N-1\right)\phi {\left(1-\phi \right)}^{N-2}\left(1-\alpha \right)P_{i-7}P_{i-3};\\ \;\;\;for\;i=9. \end{array}\right. \end{array} \end{array}$$

Therefore, the probability of finding the channel busy during the CCA is

(21) $$\begin{array}{cc} \; & \alpha =\sum_{i=1}^{9}\alpha_{i}. \end{array}$$

### 4.1. Reliability

A transmission is successful if exactly one node transmits data without any collision and channel error. A node can be successfully associated to a coordinator if the coordinator receives the association request successfully and the node receives the acknowledgement successfully. Hence, the probability that the node is associated successfully with the coordinator is

(22) $$\begin{array}{cc} P_{S_{1}} & =\frac{N\phi {\left(1-\phi \right)}^{N-1}}{\left(1-{\left(1-\phi \right)}^{N}\right)}P_{1}P_{5}. \end{array}$$

After successfully associating with the coordinator, the nodes go for the data transmission. The transmission of aperiodic data by a node is successful if exactly one node is transmitting without any collision or channel error. The equation of the probability that the node transmits aperiodic data successfully is

(23) $$\begin{array}{cc} P_{S_{2}} & =\frac{N\phi {\left(1-\phi \right)}^{N-1}}{\left(1-{\left(1-\phi \right)}^{N}\right)}P_{2}P_{6}. \end{array}$$

A node can get the GTS slot successfully if the coordinator receives the GTS request successfully and the node gets the acknowledgement along with the GTS reply packet successfully. The equation of probability that the node gets the GTS slot successfully from the coordinator is given as follows.

(24) $$\begin{array}{cc} P_{S_{3}} & =\frac{N\phi {\left(1-\phi \right)}^{N-1}}{\left(1-{\left(1-\phi \right)}^{N}\right)}P_{3}P_{7}. \end{array}$$

If a node misses the beacon, it can get its GTS information by sending the information request packet to the coordinator. The retrieval of GTS information will be successful if the coordinator receives the information request successfully and the node gets the corresponding acknowledgement along with the information response successfully. Hence, the probability of retrieving the information packet successfully from the coordinator is

(25) $$\begin{array}{cc} P_{S_{4}} & =\frac{N\phi {\left(1-\phi \right)}^{N-1}}{\left(1-{\left(1-\phi \right)}^{N}\right)}P_{4}P_{8}. \end{array}$$

The probability of getting the transmission successfully is given as follows.

(26) $$\begin{array}{cc} P_{S} & =\frac{\sum_{j=1}^{4}q_{j}P_{S_{j}}}{\sum_{j=1}^{4}q_{j}}. \end{array}$$

After successfully getting the GTS slots, the node transmits the data packets during its allocated GTS slots. Transmission in the GTS slots fails due to channel error, failure of beacon tracking or if the node does not get the GTS information before the allocated GTS slots. Thus, the probability of successful transmission in the GTS slot is

(27) $$\begin{array}{c} P_{GTS}=\left(P_{10}+\left(1-P_{10}\right)P_{S_{4}}\right){\left(1-P_{b}\left(\zeta \right)\right)}^{L_{D}} \end{array}$$

where, $L_{D}$ is the data packet length. The data packet is transmitted successfully if the node is associated and gets the GTS slots successfully. Hence, the reliability is

(28) $$\begin{array}{c} Reliabilty=P_{S_{1}}P_{S_{3}}P_{GTS}. \end{array}$$

### 4.2. Delay

When two nodes in a network communicate with each other, it takes a certain amount of time for the information to be generated by the source node and to be received by the destination node. The total time elapsed during this process is called network delay. Here, we consider only the transmission delay for transmitting the data successfully in the GTS slots. Data transmission is successful in the *j*th superframe, only if the transmission fails in the previous (j−1) superframe and no new data arrives during this period. Let $P_{j}^{S}$ be the probability that the transmission of data in the GTS slot in its *j*th superframe is successful. Therefore, $P_{j}^{S}=\left(1-\chi \right)\chi^{j}$, where χ is the probability that the transmission in the GTS slot fails and no new data frame arrives in the beacon interval BI. Thus, $\chi =\left(1-P_{GTS}\right)e^{-BI\lambda }$, where λ is the data arrival rate. Hence, the average transmission delay in the GTS slots can be expressed as follows.

(29) $$\begin{array}{c} \begin{array}{cc} \Lambda & =\sum_{j=0}^{\infty }P_{j}^{S}\left(\varsigma +jBI\right)\\ \; & +transmission\;delay+propagation\;delay \end{array} \end{array}$$

where, ς is the round trip delay

$Transmission\;delay=\frac{Payload}{datarate}$

and

$Propagation\;delay=\frac{Distance\;between\;the\;source\;and\;sink\;node.}{Propagation\;speed\;in\;medium}$.

### 4.3. Packet Drop Rate

A packet is dropped before the transmission if a new packet is available. Hence, the packet is dropped in the first superframe if there is at least one new arrival within the beacon interval. Therefore, the probability that the packet is dropped in the first superframe is $P_{d_{0}}=1-e^{-BI\lambda }$. In general, the packet is dropped at the *j*th superframe only if the transmission failed in the previous (j−1) superframe and no new data has arrived during these periods, though new data arrives in the next superframe. Therefore, the packet dropped in the *j*th superframe is

$$\begin{array}{cc} P_{d_{j}} & =\left(1-e^{-BI\lambda }\right){\left(1-P_{GTS}\right)}^{j}e^{-jBI\lambda }. \end{array}$$

Hence, the overall packet drop rate in the GTS slot is

(30) $$\begin{array}{c} P_{drop}=\sum_{j=0}^{\infty }P_{d_{j}}. \end{array}$$

### 4.4. Throughput

In this part, we analyze the throughput for the CFP period. As per our assumption, a node should get a GTS slot to transmit the data in the CFP period. When the node looses synchronization, it retrieves the information through the CSMA/CA mechanism before the scheduled GTS slot. Hence, the data packet will be successfully transmitted if the node synchronizes and transmits the data successfully during the allocated GTS slots. The throughput is expressed as the fraction of time spent in transmitting the data packet successfully. Hence, the throughput during CFP can be derived as follows.

$$\begin{array}{ll} T_{CFP} & =P_{S_{3}}P_{GTS}\times \\ & \frac{Data\;payload\times Number\;of\;GTS\;packet}{Number\;of\;GTS\;slot\;required\times GTS\;slot\;length} \end{array}.$$

### 4.5. Energy

In our analysis, energy consumption is defined as the average amount of energy consumed by a node for successful transmission of data. The energy consumption for receiving data, turning around from receiving to transmitting mode or vice versa and for transmitting a packet is taken to be $P_{RX}$, $P_{AVX}$ and $P_{TX}$, respectively. Let $T_{L}$, $T_{A}$, $T_{CCA}$, $T_{ta}$ and $\delta_{max}$ be the time duration for transmitting a packet, for receiving an acknowledgement, for successful channel assessment, for turnaround and maximum time to wait for acknowledgement, respectively. The total energy consumption per node can be analyzed as follows.

(31) $$\begin{array}{cc} E & =\frac{\alpha T_{CCA}P_{RX}+\left(1-\alpha \right)\left\{\left(1-P_{S}\right)E_{c}+P_{S}E_{s}\right\}}{\left(1-\alpha \right)P_{S}T_{L}} \end{array}$$

where, $P_{S}$ is the probability of the successful transmission, $E_{s}$ is the energy consumption of successful transmission, and $E_{c}$ is energy consumption due to collision. $E_{s}$ and $E_{c}$ can be calculated as the following equation.

(32) $$\begin{array}{c} \left.\begin{array}{cc} E_{s} & =T_{CCA}P_{RX}+T_{ta}P_{AVX}+T_{L}P_{TX}+T_{A}P_{RX}\\ E_{c} & =T_{CCA}P_{RX}+T_{ta}P_{AVX}+T_{L}P_{TX}+\delta_{max}P_{RX} \end{array}\right\} \end{array}$$

## 5. Performance Evaluation

In this section, we validate and evaluate our model by using the OMNeT++ [39] simulator. In this paper we consider the mesh topology, where nodes are randomly located in the area of 500 × 500 m${}^{2}$. We further compare our proposed protocol with the IEEE 802.15.4e standard for the probability of successful transmission, throughput, reliability, average MAC delay and packet drop rate over the Poisson arrival rate. The simulation parameters are set as per the IEEE 802.15.4e standard and are provided in Table 4.

**Figure 5:**
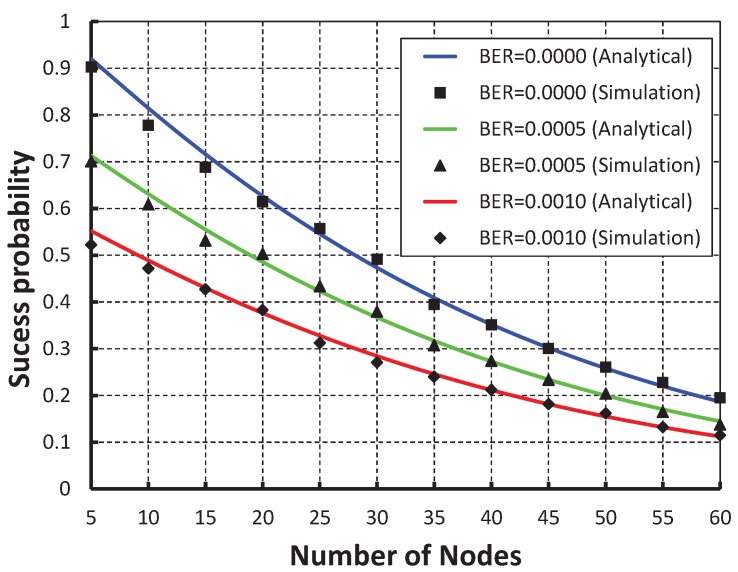
Validation of packet success for different sensor nodes.

As shown in Figure 5, the number of nodes are taken along the X axis and the corresponding success probability of packet transmission is obtained as shown in the Y axis. It is observed that the packet success probability decreases as the number of nodes attached to a coordinator increases irrespective of the bit error rate. When the data payload is 100 bytes and five nodes are considered, the successful probability is 0.91, 0.74 and 0.56 for BER (bit error rate) 0, and 0.0005 and 0.0010, respectively.

**Figure 6:**
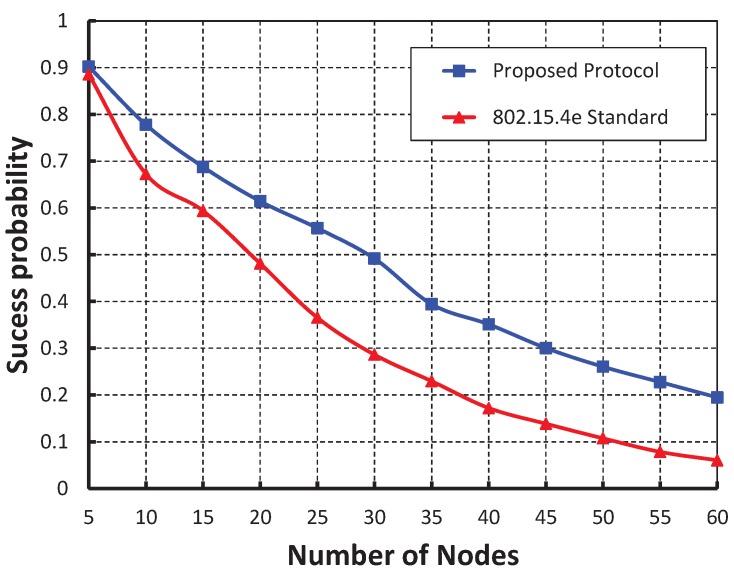
Comparison of packet success of our proposed protocol with the IEEE 802.15.4e standard.

As shown in Figure 6, we compared our work with IEEE the 802.15.4e protocol and it is observed that packet success probability of our work is higher and our simulation result as shown in Figure 5 matches the analytic one.

**Figure 7:**
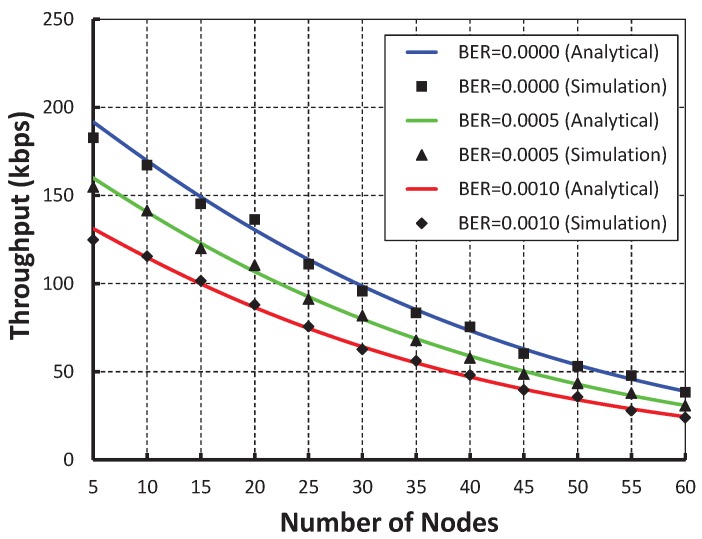
Validation of throughput for different sensor nodes.

**Figure 8:**
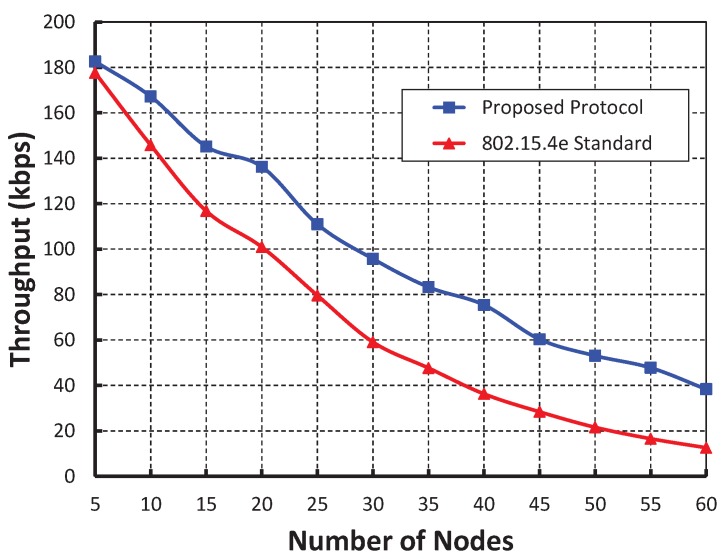
Comparison of throughput of our proposed protocol with the IEEE 802.15.4e standard.

Figure 7 demonstrates the throughput corresponding to the number of nodes. We observe that the throughput decreases as the number of nodes attached to a coordinator increases irrespective of variable bit error. When the data payload is 100 bytes and five nodes are attached to the coordinator, the throughput is 191 Kbps if there is no bit error. However, the throughput is 155 and 127 Kbps for BER 0.0005 and 0.0010, respectively. Figure 8 shows that when we studied our work with the IEEE 802.15.4e standard protocol, it is observed that the throughput of our work is higher.

**Figure 9:**
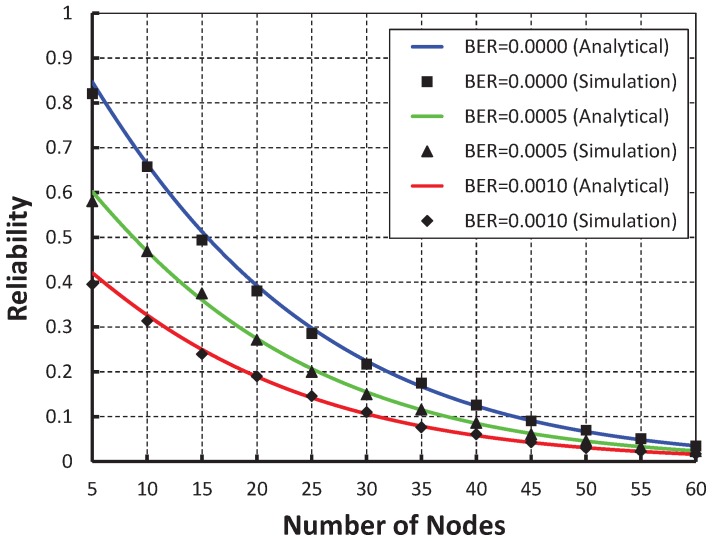
Validation of reliability for different sensor nodes.

**Figure 10:**
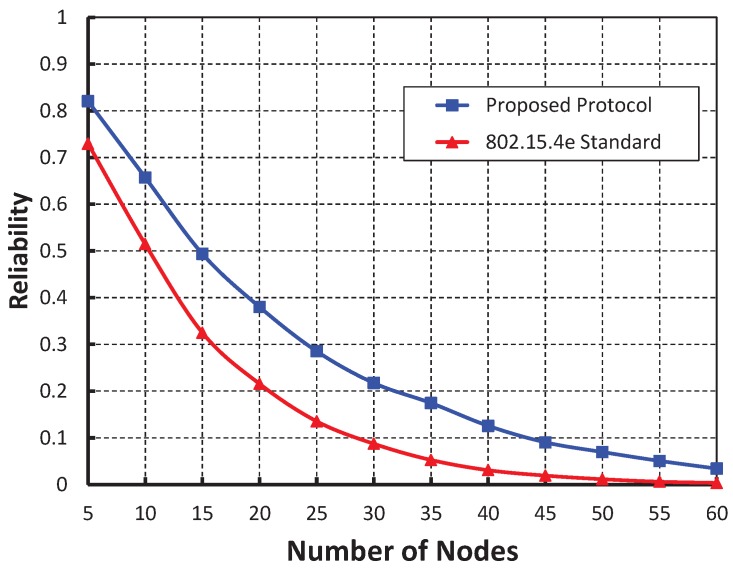
Comparison of reliability of our proposed protocol with the IEEE 802.15.4e standard.

Figure 9 compares the reliability of data transmission with respect to different nodes. It is observed that the reliability decreases as the number of nodes attached to a coordinator increases irrespective of the bit error. When the data payload of 100 bytes and five nodes are considered, the reliability is 0.84, if there is no bit error rate. However, 0.60, 0.42 for the BER is 0.0005 and 0.0010, respectively. From Figure 10, it is observed that the reliability of our work is higher than that of the IEEE 802.15.4e protocol and our simulation results match well with the analytic ones.

**Figure 11:**
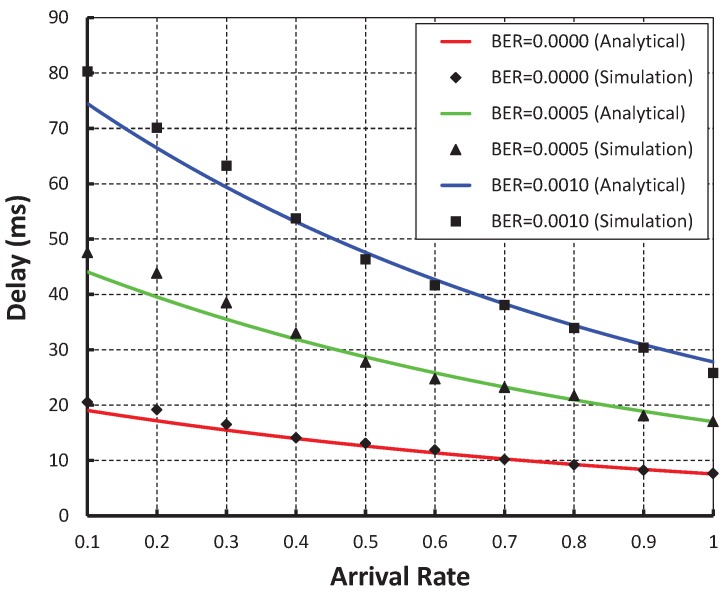
Validation of packet delay with arrival rates.

**Figure 12:**
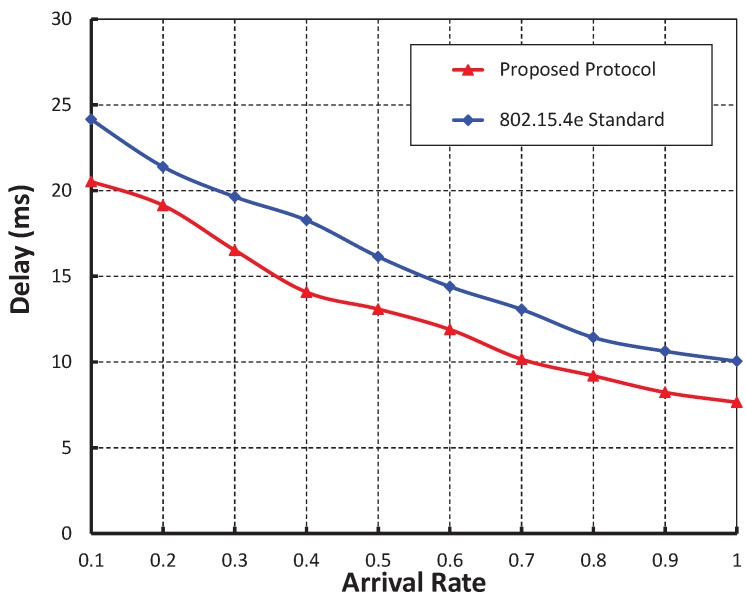
Comparison of packet delay of our proposed protocol with the IEEE 802.15.4e standard.

As shown in Figure 11, the arrival rate is taken along the X axis and the corresponding delay is obtained in the Y axis. It is observed that the delay decreases as the arrival rate increases irrespective of the bit error rate. When the data payload is 100 bytes and twenty nodes are considered the delay is nearly 20 ms if there is no bit error, whereas, the delay is nearly 47 ms and 79 ms for BER 0.0005 and 0.0010, respectively. From Figure 12, it can also be observed that the packet transmission delay in our work is less as compared to the IEEE 802.15.4e protocol and our simulation result coincides with the analytic one.

**Figure 13:**
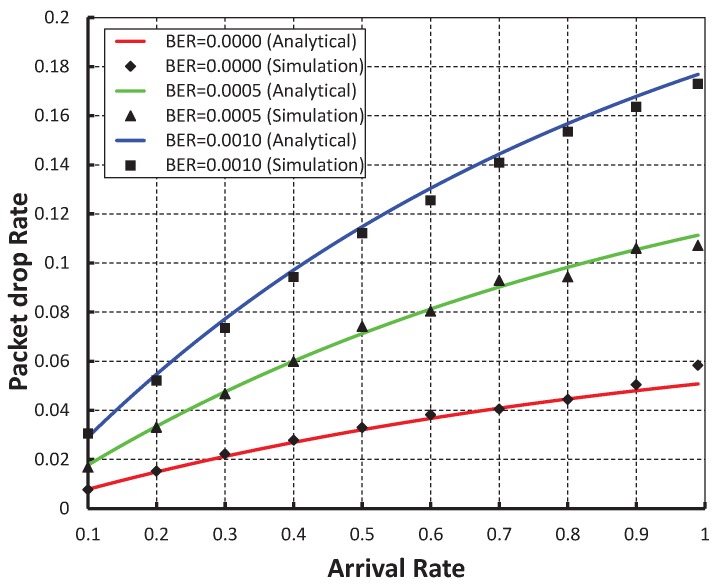
Validation of packet drop rate with arrival rates.

**Figure 14:**
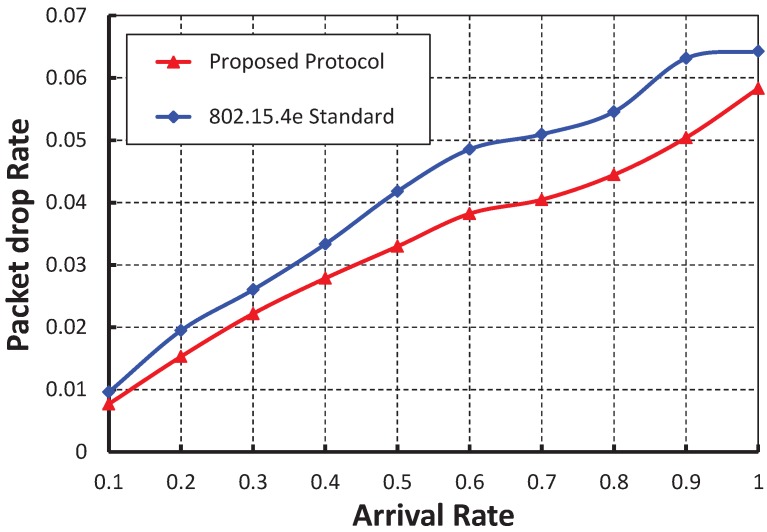
Comparison of packet drop rate of our proposed protocol with the IEEE 802.15.4e standard.

Figure 13, shows the packet drop rate with variable arrival rate. It is observed that the delay decreases as the arrival rate increases irrespective of the bit error rate. When the data payload is 100 bytes and twenty nodes are considered, the packet drop rate is nearly 0.004 if there is no bit error. However, the packet drop rate is nearly 0.009 and 0.015 for the BER 0.0005 and 0.0010, respectively. As shown in Figure 14, the packet drop rate of our work is less than the IEEE 802.15.4e protocol.

**Figure 15:**
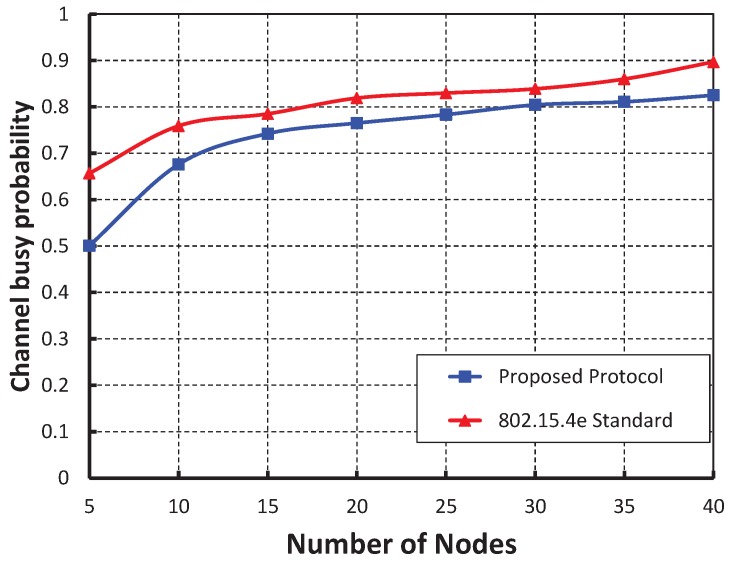
Comparison of channel busy of our proposed protocol with the IEEE 802.15.4e standard.

As shown in Figure 15, the Y axis is the channel busy probability and the X axis is the different number of nodes. The channel busy probability increases with respect to the number of nodes. We also found that the channel busy probability of our work is less than that of the IEEE 802.15.4e protocol.

**Figure 16:**
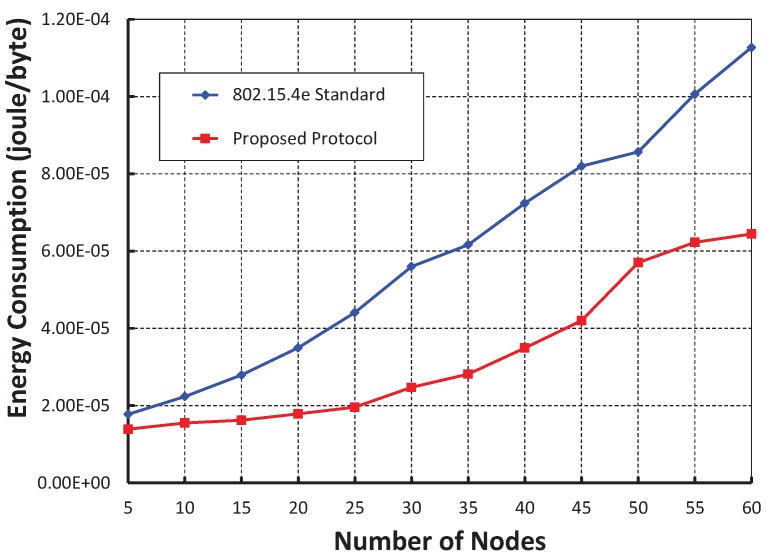
Comparison of energy consumption (*E*) of our proposed protocol with the IEEE 802.15.4e standard.

Figure 16 shows the energy consumption in joule/byte with different numbers of nodes. It is observed that the energy consumption of our work is significantly less than the IEEE 802.15.4e protocol. We achieve this result through our proposed single CCA operation rather than two times consecutive CCA operations, as suggested in the IEEE 802.15.4e standard, before any data transmission and by taking an appropriate sleep period when the channel is found to be busy during CCA.

## 6. Conclusions

In this paper, we propose a new channel access mechanism, which can significantly reduce the power consumption during the communications in control channel. An LPP model is designed to select the relay nodes, which can reduce the delay and packet drop rate among the nodes in a mesh topology as compared to the current IEEE 802.15.4e standard. Analytical models are designed to study the performance metrics of WSNs such as data transmission reliability, throughput, energy consumption and delay. The results obtained from the simulation indicate that our protocol can improve the energy, reliability, throughput and packet success rate significantly. Hence, our protocols can be implemented in synchronous MAC based WSNs for industrial, commercial and healthcare applications, where energy, latency, reliability, scalability and drop rate are the major requirements.

## Figures and Tables

**Table 1:** Comparison of related works.

Algorithm	Type of Network	MAC Type	Channel Type	Hop Type	Analysis	Contributions	Limitations
SHARE [1]	Hetro.	IEEE 802.22	Multi	Multi	Throughput, collision	Reduces packet collisions	Not suitable for low power sensors
TRM-MAC [4]	Tree	IEEE 802.15.4	Single	Single	Latency, reliability, energy	Improves reliability	Not suitable for multi-hop network
M−CSMA /CA [5]	Star	IEEE 802.15.4e	Single	Single	Reliability, throughput, energy, delay	Reduces the packet drop rates	Single hop network
C-LLF [7]	Mesh	Wireless HART	Single	Multi	Delay	End-to-end real-time transmission	Lacks performance analysis
C-LLF [8]	Mesh	Wireless HART	Single	Multi	Delay	Scheduling of real-time data flows	Lacks performance analysis
ORST [10]	Mesh	IEEE 802.15.4	Single	Multi	Energy	Improve communication reliability	Only for periodic data
ANCAS [31]	Mesh	IEEE 802.15.4e	Single	Single	Reliability, delay	Reduces GTS retransmission delay	Not suitable for multi-hop network
Our Protocol	Mesh	IEEE 802.15.4e	Multi	Multi	Reliability, throughput, energy, delay, packet drop rate	Enhances channel access mechanism of IEEE 802.15.4e DSME MAC, Evaluates most performance metrics	Suitable for two hop communication

**Table 2:** Symbols with meaning.

Notation	Meaning
*N*	Number of nodes present within carrier sensing range;
*L*	Length of the data packet;
*p*	Insufficient time probability for transmission;
$P_{b}\left(\zeta \right)$	Bit error rate probability;
$P_{S}$	Transmission success probability;
λ	Data arrival rate;
μ	Data transmission rate;
BF	Buffer capacity of a node;
RE	Residual energy of a node;
ϕ	Probability that a node is having 1st CCA;
α	Probability of finding a channel busy.

**Table 3:** Notation table.

Notation	Meaning
$L_{1}$	Length of the association packet;
$L_{2}$	Length of the aperiodic data packet;
$L_{3}$	Length of the GTS request packet;
$L_{4}$	Length of the information request packet;
$L_{5}$	Length of the association reply packet;
$L_{6}$	Length of acklodgement to aperiodic data packet;
$L_{7}$	Length of the GTS request reply packet;
$L_{8}$	Length of the information request reply packet;
$L_{9}$	Length of the GTS request notify packet;
$L_{10}$	Length of the beacon packet;
$q_{1}$	Generation probability of the association packet;
$q_{2}$	Generation probability of the aperiodic data packet;
$q_{3}$	Generation probability of the GTS request packet;
$q_{4}$	Generation probability of the information request packet.
$P_{S_{1}}$	Probability of association success;
$P_{S_{2}}$	Probability of aperiodic data transmission successful;
$P_{S_{3}}$	Probability of getting GTS slot successfully;
$P_{S_{4}}$	Probability of information retrieval successful;
$P_{GTS}$	Probability of GTS transmission successful.

**Table 4:** Simulation parameters.

Parameters	Value
Radio band	2.4 GHz
Channel bandwidth	250 Kbps
Carrier sense sensitivity	−85 dBm
Channel number	11
Beacon interval	$15.16\times 2^{5}$ ms
Unit backoff period	20 symbol
PHY overhead	6 byte
MAC overhead	3 byte
Transmission current consumption	9.1 mA
Receiving current consumption	5.9 mA
Turnaround current consumption	7.5 mA
Sleep current consumption	0.001 mA
